# Assessing the Impact of PM_2.5_-Bound Arsenic on Cardiovascular Risk among Workers in a Non-ferrous Metal Smelting Area: Insights from Chemical Speciation and Bioavailability

**DOI:** 10.1021/acs.est.3c10761

**Published:** 2024-05-02

**Authors:** Zenghua Qi, Qiting Zhao, Zixun Yu, Zhu Yang, Jie Feng, Pengfei Song, Xiaochong He, Xingwen Lu, Xin Chen, Shoupeng Li, Yong Yuan, Zongwei Cai

**Affiliations:** † Guangdong-Hong Kong-Macao Joint Laboratory for Contaminants Exposure and Health, School of Environmental Science and Engineering, Institute of Environmental Health and Pollution Control, Guangdong University of Technology, Guangzhou 510006, China; ‡ State Key Laboratory of Environmental and Biological Analysis, Department of Chemistry, Hong Kong Baptist University, Kowloon 999077, Hong Kong, China; § The Center for Reproductive Medicine, Shunde Hospital, Southern Medical University (The First People’s Hospital of Shunde), 528300 Foshan, Guangdong, China; ∥ Analysis and Test Center, Guangdong University of Technology, Guangzhou 510006, China

**Keywords:** PM_2.5_-As, Chemical speciation, Bioavailability, Heart dysfunction, Health risk assessment

## Abstract

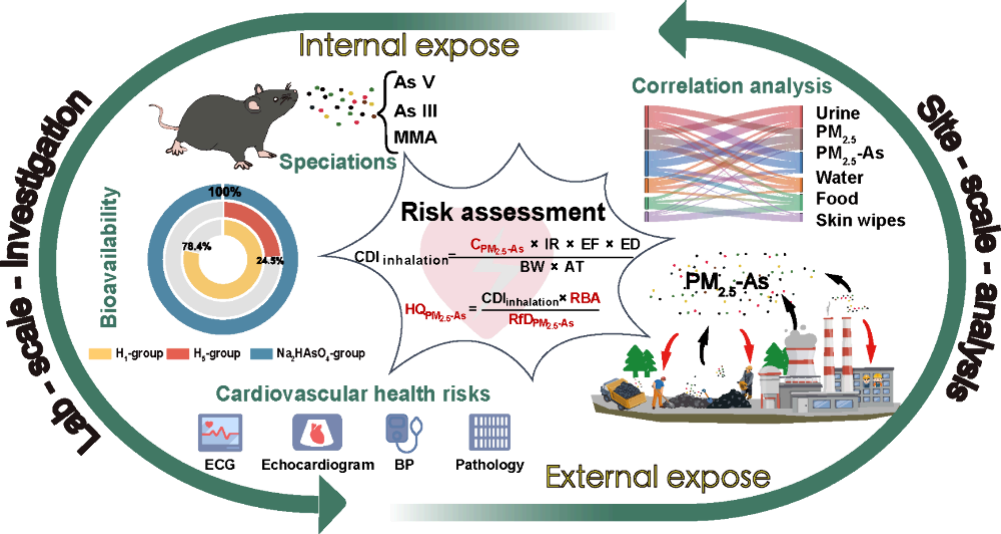

Inhalation of fine particulate matter PM_2.5_-bound arsenic (PM_2.5_-As) may cause significant cardiovascular damage, due to its high concentration, long transmission range, and good absorption efficiency in organisms. However, both the contribution and the effect of the arsenic exposure pathway, with PM_2.5_ as the medium, on cardiovascular system damage in nonferrous smelting sites remain to be studied. In this work, a one-year site sample collection and analysis work showed that the annual concentration of PM_2.5_-As reached 0.74 μg/m^3^, which was 120 times the national standard. The predominant species in the PM_2.5_ samples were As (V) and As (III). A panel study among workers revealed that PM_2.5_-As exposure dominantly contributed to human absorption of As. After exposure of mice to PM_2.5_-As for 8 weeks, the accumulation of As in the high exposure group reached equilibrium, and its bioavailability was 24.5%. A series of animal experiments revealed that PM_2.5_-As exposure induced cardiac injury and dysfunction at the environmental relevant concentration and speciation. By integrating environmental and animal exposure assessments, more accurate health risk assessment models exposed to PM_2.5_-As were established for metal smelting areas. Therefore, our research provides an important scientific basis for relevant departments to formulate industry supervision, prevention and control policies.

## Abstract 2

Combined with the internal and external exposure parameters, we determined the dominant contribution of PM_2.5_-As exposure for human absorption along with a more scientific and accurate health risk assessment model.

## Introduction

In relation to nonferrous metals, China is both the world’s largest producer and consumer. In 2022, 67.7 million tons of ten nonferrous metals were produced, an increase of 4.30% year-on-year.^1^ However, heavy metal pollution in the nonferrous metal smelting industry has been a prominent environmental concern in China.^2^ Over recent years, with advances in the smelting process and environmental supervision, the exposure pathways and contribution weightings of heavy metals associated with smelting sites are likely to have undergone changes. Hence, according to the existing smelting technology and control measures, identifying the main exposure pathways of heavy metals associated with smelting sites and elucidating the ultimate health effects of heavy metal exposure are not only practical requirements for over 2 million employees and residents, but also hotspots and challenges for research in the fields of earth environmental science, epidemiology, and life sciences.

Arsenic (As) has been proven to induce various CVDs such as coronary heart disease,^3^ peripheral arterial disease,^4^ stroke,^5^ arrhythmia,^6^ and hypertension.^7^ Heavy metal As itself has cardiotoxicity, while fine particulate matter (PM_2.5_) carrying heavy metal As (PM_2.5_-As) in nonferrous metal smelting areas has the characteristics of diverse species, high concentration, long transmission distance in the atmosphere and high absorption rate in organisms, which may cause greater cardiovascular damage.^8−11^ According to our previous study, approximately 25.0% of workers from smelting areas exhibited abnormal cardiopulmonary indexes, suggesting an increased risk of cardiovascular health issues among workers in these areas.^9^ However, the contribution, effects, and toxic mechanisms of the arsenic exposure pathway through PM_2.5_ in nonferrous smelting sites to cardiovascular system damage are still in need of further research.

In the past few years, researchers have gradually realized that it is hard to assess the health risks of environmental pollutants exposure accurately by traditional assessment models using single pollution indices such as exposure mass, exposure pathways, and nontargeted health reference dose (RfD).^12,13^ The chemical speciations of heavy metals and their related bioavailability are also essential factors in the evaluation of their health hazards.^9,14^ Inorganic As in atmospheric particles are the main species, while trace amounts of MMA have also been found.^15^ The toxic effect of arsenic is greatly determined by the chemical forms and possible molecular structure. As (III) and As (V) are considered the most highly toxic species, whereas organic compounds such as arsenobetaine (AsB), which are formed through the methylation of inorganic species (known carcinogens), are comparatively less toxic.^16,17^

There is a lack of solid clinical evidence on the threat of PM_2.5_-As to people’s health in nonferrous metal smelting industry. However, its high concentrations and the unambiguous evidence of its cardiovascular toxicity in animals indicate that it is essential to understand better the cardiovascular risk to workers in the smelting sites from the perspective of As speciation and bioavailability.

To this end, we first clarified the contamination profile and chemical speciation of PM_2.5_-As in a nonferrous metal smelting area. Subsequently, a comprehensive epidemiological analysis and animal inhalation exposure studies depended on environmentally relevant exposure level and chemical speciation was carried out to explore the contribution of PM_2.5_-As exposure for human absorption along with its relative bioavailability (RBA) and adverse effects on the heart. Finally, we established the more scientific and accurate health risk assessment models of PM_2.5_-As exposure in the smelting sites from the perspective of As speciation and bioavailability.

## Materials and Methods

### Study Site and Sample Collection

This research site is a typical lead–zinc smelter, covering lead–zinc ore grinding, smelting and sulfuric acid extraction. The factory is located in Gansu Province, China’s main nonferrous metal production base and has been operating for 45 years. The factory covers 27.5 km^2^, and there are about 3000 employees. The factory is at a distance from any urban area, and there are no other factories or obvious pollution sources around it, thus ensuring the independence of the environmental samples taken in this study and providing clearer and more accurate environmental background and data.

Representative PM_2.5_ samples were collected according to the requirements of Ambient Air Quality Standards (GB3095–2012, Figure S1). All samples were collected every third day using a fine particulate matter sampler (Laoying Co. Ltd., Qingdao, China) at a flow rate of 100 L/min for a whole day, fitted with Whatman QMA quartz microfiber filters (Φ90 mm, Maidstone, England). After sampling and weighing, the particulate matter mass was calculated by deducting the filter mass and then storing them using the same procedure as prior to analysis (Text S1). An average of 12 samples were collected from the smelting site every month, yielding a total of 96 valid samples of PM_2.5_ collected over four seasons.

### Determination of Total Arsenic

After being divided into pieces, half of the PM_2.5_ filters were put in a sample-dissolving tube and moistened with ultrapure water. In a fume hood, 6 mL of 30% HCl was initially added, with 2 mL of 68% HNO_3_ then gradually added. It was ensured that the sample was brought into complete contact with the digestion solution. After 5 min, the tubes were inserted into the digestion tank for sealing, and both were then placed into the furnace chamber of the microwave dissolver (MARS6, CEM Co. Ltd., USA). The heating program was set up according to Table S1. After the program was completed, the tubes were allowed to cool off.

The digested solution was filtered through a 0.22 μm aqueous phase polyether sulfone needle filter, the digestion tube and precipitate were washed with 2% HNO_3_, and all the solutions were incorporated into a 25 mL volumetric flask. Finally, 2% HNO_3_ was added to constant volume to the line and mixed well. A suitable quantity of the internal standard mixture (^45^Sc, ^73^Ge, ^89^Y, ^115^In, ^185^Re, ^193^Ir, and ^209^Bi) was then added. The solution was mixed before being characterized using inductively coupled plasma-mass spectrometry (ICP-MS; model iCAP RQ, Thermo Fisher Scientific, USA). Comprehensive details regarding total arsenic (tAs) detection can be found in the Supporting Information (Text S2 and Table S2).

### Arsenic Speciation Analysis

We carried out an analysis of the chemical speciation of PM_2.5_-As from a chemical perspective. X-ray photoelectron spectroscopy (XPS) analysis was carried out using an X-ray photoelectron spectrometer (Escalab 250 Xi, Thermo Fisher Scientific Co. Ltd., USA) under an ultrahigh vacuum (about 10^–7^ Pa). By utilizing the internal standard of the added carbon and its C (1s) binding energy (284.8 eV) for charge compensation, we acquired and rectified the narrow scan spectra of As (3d). XPS analysis process and detection parameters are given in Supporting Information (Text S3, Table S3).As with PM_2.5_, samples were extracted using ultrapure water. The chemical speciation of As was examined using liquid chromatography and atomic fluorescence spectrometry (ELSpe-2, Prin-Cen Co., Ltd., Guangzhou, China). Arsenicals were separated with a separation column (4 mm × 50 mm, Prin-Cen arsenic speciation rapid analysis column, Prin-Cen Co., Ltd., Guangzhou, China), and the concentrations were quantified using the AFS system with standard calibration curves.

### Study Population and Sample Collection in the Panel Study

In order to clarify the main path of As exposure of workers at the site, we carried out panel studies in summer and winter to analyze the correlation between As in workers’ urine and the main exposure media of the smelting site. In the panel study, employees at the sample location answered a thorough questionnaire before sampling, including basic data, operational positions, daily routines, habits, medical histories, and symptoms of CVD. Using the questionnaire, we screened 25 workers who were healthy, nonsmokers, had no important pollution sources around their living areas and had worked continuously in the factory for 1 year, as the study population. The operation process of this cohort study was approved by the Ethical Committee of Shunde Hospital, Southern Medical University (KYLS20220127).Environmental samples and human samples of each volunteer are collected continuously for 15 days per season, and the collection time and frequency are completely in accordance with the workers’ work schedules. The different kinds of food purchased from the staff canteen and drinking water obtained from the staff’s lounge and office were used as samples of their daily diet. At the conclusion of their daily work, each participant utilized Ghostwipes (SC4250, PA, U.S.A) to wipe the palms and dorsa of their hands, along with their foreheads, to give samples of skin exposure. Moreover, participants were required to collect their urine at least once while at work. If they collected more than one sample then the urine samples were mixed. Following sample collection, each sample was placed in 50 mL Teflon tubes, put straight into the refrigerator at −20 °C, then placed in dry ice before being delivered to the laboratory for further analysis. The corresponding PM_2.5_ samples were also collected at the same time, as described in Section Study Site and Sample Collection.

Food samples were weighed into 0.5000 ± 0.0005 g portions after being freeze-dried for 48 h; 2 mL of drinking water samples and urine samples were taken separately, and 1 cm^2^ of skin contact samples were cut into pieces. The tAs in food, water and human samples were measured using the ICP-MS described in Section Determination of Total Arsenic.

### Internal Dose and Cardiovascular Toxicity Assessments for Exposure to PM_2.5_-As in Mice

To replicate the real As exposure scenario in PM_2.5_ at the smelting site as closely as possible, mixed exposure solutions based on the concentration and form of PM_2.5_-As in the real environment determined through analysis of filter membranes were used for animal inhalation exposure. After being acclimatized (7 days), fifty-six healthy male C57BL/6 mice, aged 6 weeks, were randomized and assigned into seven groups (8 mice for each group) for carrying out the exposure experiments. The seven groups were: two as low concentration groups (L groups, including a L_1/25_ group of 0.06 μg/(kg·b.w.) and a L_1/5_ group of 0.31 μg/(kg·b.w.), to represent 1/25 and 1/5 respectively of the medium concentration of PM_2.5_-As collected at the sampling site), one as a medium concentration group (M group, 1.57 μg/(kg·b.w.), to represent the medium concentration of PM_2.5_-As), two as high concentration groups (H groups, including a H_1_ group of 8.51 μg/(kg·b.w.) and a H_5_ group of 42.5 μg/(kg·b.w.), to represent respectively the maximum concentration of PM_2.5_-As and 5-fold maximum concentration of PM_2.5_-As), one soluble reference group (Na_2_HAsO_4_ group, 8.51 μg/(kg·b.w.), equal to the maximum concentration of PM_2.5_-As) and one control group (C group, an equal dose of saline).

The calculation method and data for the simulated exposure solution of different groups can be found in Text S4, S5 and Table S4. Mice of all groups received intratracheal instillation of 20 μL of the corresponding exposure solution (arsenic mixed solutions, saline, and Na_2_HAsO_4_) three times per week. The instillation process was repeated three times per week until clear cardiovascular damage was detected, or until the concentrations of As in urine reached a stable state, indicating an equilibrium between As absorption and metabolism in the mice. During the exposure period, body weight, HR, and physical condition were noted weekly. Urine and feces were also collected weekly, and blood glucose was assessed every 2 weeks. At the end of the exposure, BP and echocardiographic examination (Vevo 2100, Fujifilm, Visual Sonics, Toronto, Canada) were carried out according to our previously published methods^9,18^ with slight modifications. 8-OHdG detection in the urine and blood of As-exposed mice was carried out using an 8-OHdG ELISA kit (Sangon Biotech Co. Ltd., Shanghai, China). The detailed procedures for H&E staining and Masson staining are provided in the Supporting Information (Text S6). This animal study was approved by the Animal Ethics Committee of Guangdong University of Technology.

### As Bioaccumulation, Speciation and Bioavailability Analysis

The heart, stomach, liver, spleen, lung and aorta were taken out from a −80 °C ultralow temperature refrigerator and immediately vacuum freeze-drying for 2 days. The tissue was then weighed, processed, and crushed in a homogenizer (JXFSTPRP-24, Jingxin Co. Ltd., China) at 4 °C, using zirconia grinding beads. The materials were transferred into 6 mL 30% HCl and 2 mL 68% HNO_3_ for predigestion. The arsenic concentration in hair was measured at the same time. Microwave digestion and tAs concentration detection were carried out according to the procedures outlined in Section Determination of Total Arsenic.

The chemical speciation of As in tissues and urine was identified by adapting the procedure used in previous studies.^19−21^ After being ground by liquid nitrogen, 300 mg of tissue samples were weighed and homogenized using the grinder. After homogenization, samples were incubated with 20 mL HNO_3_(0.15 mol/L) for 2.5 h at 90 °C. The supernatant was filtered through a 0.45 μm filter. The 5 mL urine sample was centrifuged at 4500 rpm for 10 min, and filtered with a 0.45 μm filter for direct chemical analysis. Four As species (As (III), As (V), MMA and DMA) in extracts or urine were separated and identified via a PerkinElmer 350X ICP-MS (PerkinElmer, USA) connected to a HPLC unit (Prin-Cen ELSpe-2 HPLC, Prin-Cen Scientific, China). HPLC was equipped with a guard column (4 × 50 mm, IonPac AG19) and a separation column (4 × 250 mm, IonPacAS19). The mobile phase was 50 mmol/L ammonium carbonate with a flow rate of 1.0 mL/min and pH = 9.5. The column temperature was set at 25 °C. The concentration of each arsenic speciation was obtained by converting the total amount of tissue extraction and the dilution multiple.

A linear correlation was reported between the dose of As and the accumulation of As in mice exposed to simulated inhalation.^22^ The RBA of PM_2.5_-As was calculated using the dose–effect curve as a guide. The calculation formula is as follows (eq 1):^23,24^
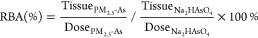
1RBA (%) is the relative bioavailability of As in mice under inhalation exposure. Tissue_PM_2.5_-As_ and Tissue_Na_2_HAsO_4__ represents the accumulation levels of PM_2.5_-As and sodium arsenate, respectively, and Dose_PM_2.5_ - As_ and Dose_Na_2_HAsO_4__ are the total inhalation dose of PM_2.5_-As and sodium arsenate on mice, respectively.

### Health Risk Assessment Modeling

Chronic daily intake (CDI) of PM_2.5_-As into the body by inhalation pathway was calculated using the health risk model provided by the U.S. Environmental Protection Agency (USEPA) (eq 2):^25^
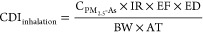
2where C_PM_2.5_ - As_ represents the concentration of As in PM_2.5_ (mg/m^3^), IR is the inhalation rate of PM_2.5_ (15.2, m^3^/d), ED is the exposure duration (24, year), EF is the exposure frequency (365 days per year), BW is the body mass (60, kg), and AT is the average exposure time through inhalation for noncarcinogenic risk (ED × 365, days) and carcinogenic risk (70 × 365, days), respectively.

To investigate the dose–effect relationship, the Benchmark Dose Tools (BMDS) Online developed by the USEPA was used to match the response of exposure dose to *in vivo* toxicological exposure data (https://bmdsonline.epa.gov/). The benchmark response (BMR) was the mean response change of 10% in comparison to the C group, and the optimal model was selected based on P value and the lowest Akaike information criterion (AIC). The bilateral 90% confidence interval of BMD (corresponding to the predefined BMR) was calculated to determine the benchmark dose (lower confidence limit) (BMDL, fifth percentile) and benchmark dose (upper confidence limit) (BMDU, 95th percentile).

To ensure precise health risk assessment, it is crucial to take into account the correlation between the Reference Dose (RfD) for chronic inhalation exposure and the concentration of the specific pollutants of interest. If the toxicity data conforms to the dose–response model, the BMDL can be used as the preferred method to determine the point of departure (POD) for the risk assessment, otherwise the no-observed-adverse-effect level (NOAEL) or the lowest observed adverse effect level (LOAEL) can be selected as the POD, so as to calculate the RfD value corresponding to the minimum effective concentration. Due to absorption not being equal to intake, we ultimately chose two internal exposure parameters RBA and RfD to adjust the Health Risk Assessment. This allowed the model to estimate appropriately the health risk to workers in the smelting region of PM_2.5_-As to the human cardiovascular system.

### Statistical Analysis

GraphPad Prism 9.0 and Origin 2021 were used for the statistical analysis, and the findings are provided as mean values ± SD. An unpaired *t* test was used to analyze the statistical significance of differences between two experimental groups, while a one-way ANOVA test was used to assess the significance of differences between three or more groups. For all statistical studies, the statistical significance level was fixed at 0.05.

## Results and Discussion

### Pollution Characteristics and Chemical Speciation of PM_2.5_-As at Smelting Sites

**Figure 1:**
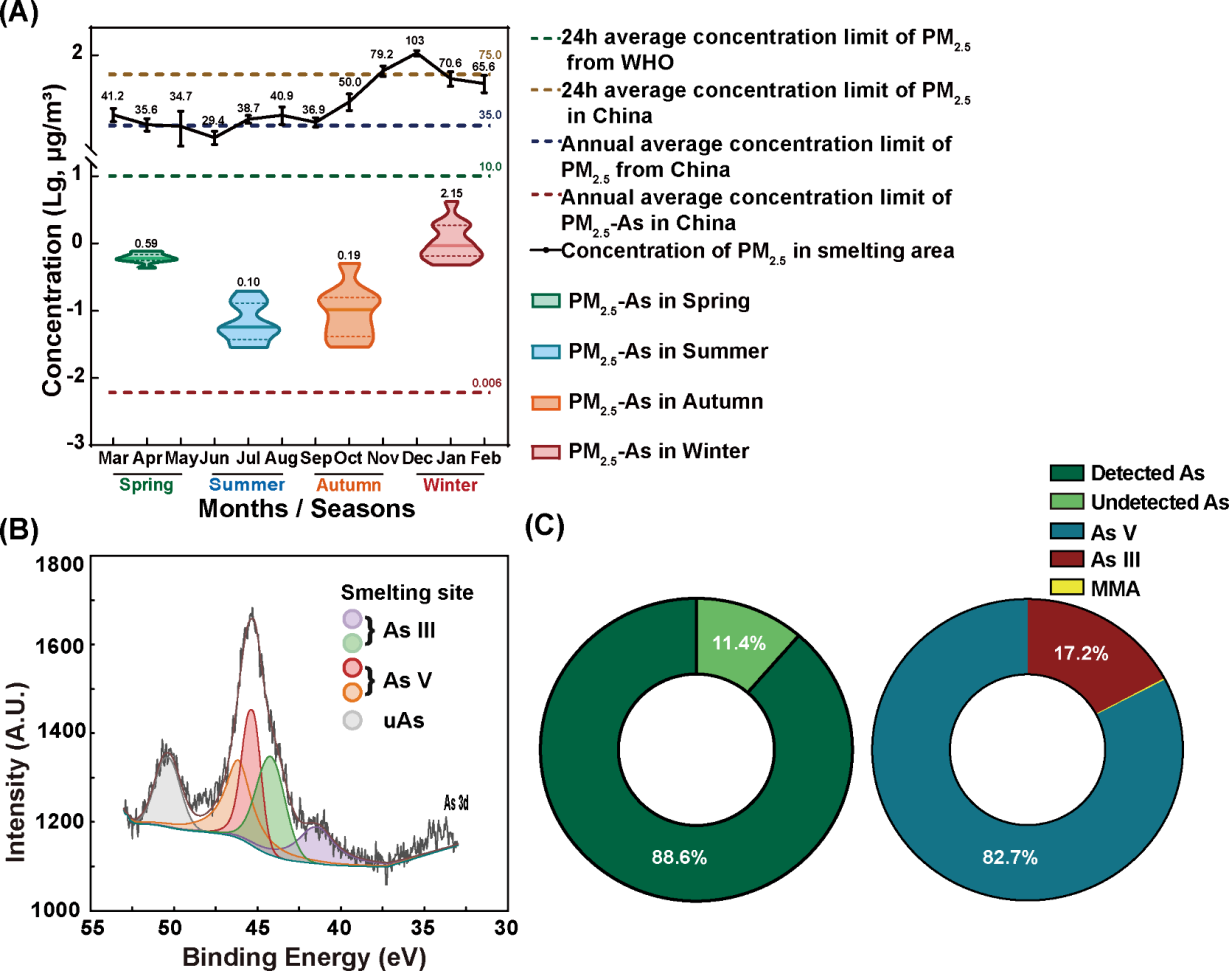
PM_2.5_-As concentration and chemical speciation analysis. (A) Concentration of PM_2.5_ and PM_2.5_-As at the smelting site from 2021 to 2022. (B) XPS spectra of As (3d) in PM_2.5_-As from the smelting site. (C) Proportion of PM_2.5_- As from LC-AFS analysis.

Monthly fluctuations in PM_2.5_ concentration and seasonal changes in PM_2.5_-As concentration from 2021 to 2022 are both shown in Figure 1A. Total PM_2.5_ concentrations from the smelting site in this study varied greatly, from 29.4 to 103 μg/m^3^, with an average of 52.2 μg/m^3^, exceeding the annual average limit in China (35.0 μg/m^3^, Ambient air quality standards, GB3095–2012). Winter had the highest levels, whereas summer had the lowest; such differences were consistent with most regions and cities in China.^18,26^

The seasonal levels in PM_2.5_-As essentially followed the trend in PM_2.5_ and reached the peak in winter at 4.00 μg/m^3^ (Figure 1A). PM_2.5_-As had an annual concentration of 0.74 μg/m^3^, which is 120 times higher than the PM_2.5_-As level set by China’s ambient quality standards. Table S5 presents a comparison of PM_2.5_-As levels in major Chinese cities. The mean value of PM_2.5_-As detected in factories is 34 times higher than the nationwide average concentration in cities. Additionally, excessive concentrations of As were also detected in the soil, dust, concentrate and tailings in the factory and surrounding areas (Figure S2), which were considered as the main potential sources of PM_2.5_-As at smelting sites.^27,28^ Due to the differences in sources, genetic mechanisms and formation time of PM_2.5_ in different regions, the pollutants carried by PM_2.5_, especially heavy metals, are also quite different in species and chemical forms. Our study investigated the speciation of PM_2.5_-As at a lead–zinc smelter using XPS. The XPS narrow-scan spectra of As (3d) in PM_2.5_-As from the smelting site are shown in Figure 1B. After peak fitting, five fitted peaks could be seen. In this case, we have reason to believe that the binding energies of 41.3 and 44.3 eV correspond to As (III) species (As_2_O_3_ = 44.1 eV), and that of 45.5 and 46.2 eV are thought to belong to As (V) species (As_2_O_5_ = 45.6 eV).^29^ This might indicate that the predominant species in the PM_2.5_ samples were As (V) and As (III).

As has a range of poisonous and harmless forms, and the toxicity relies on its chemical speciation. As (V) is the predominant species of PM_2.5_-As. Figure 1C shows the speciation of As in PM_2.5_ detected using LC-AFS, including As (V), As (III), MMA and DMA, which accounted for 88.6% of the tAs concentration in PM_2.5_ samples. The ratio of As (V), As (III) and MMA was 82.7:17.2:0.17, while DMA was not detected in PM_2.5_ samples.

### PM_2.5_-Bound As Exposure was Strongly Associated with the Human Absorption of As

**Figure 2:**
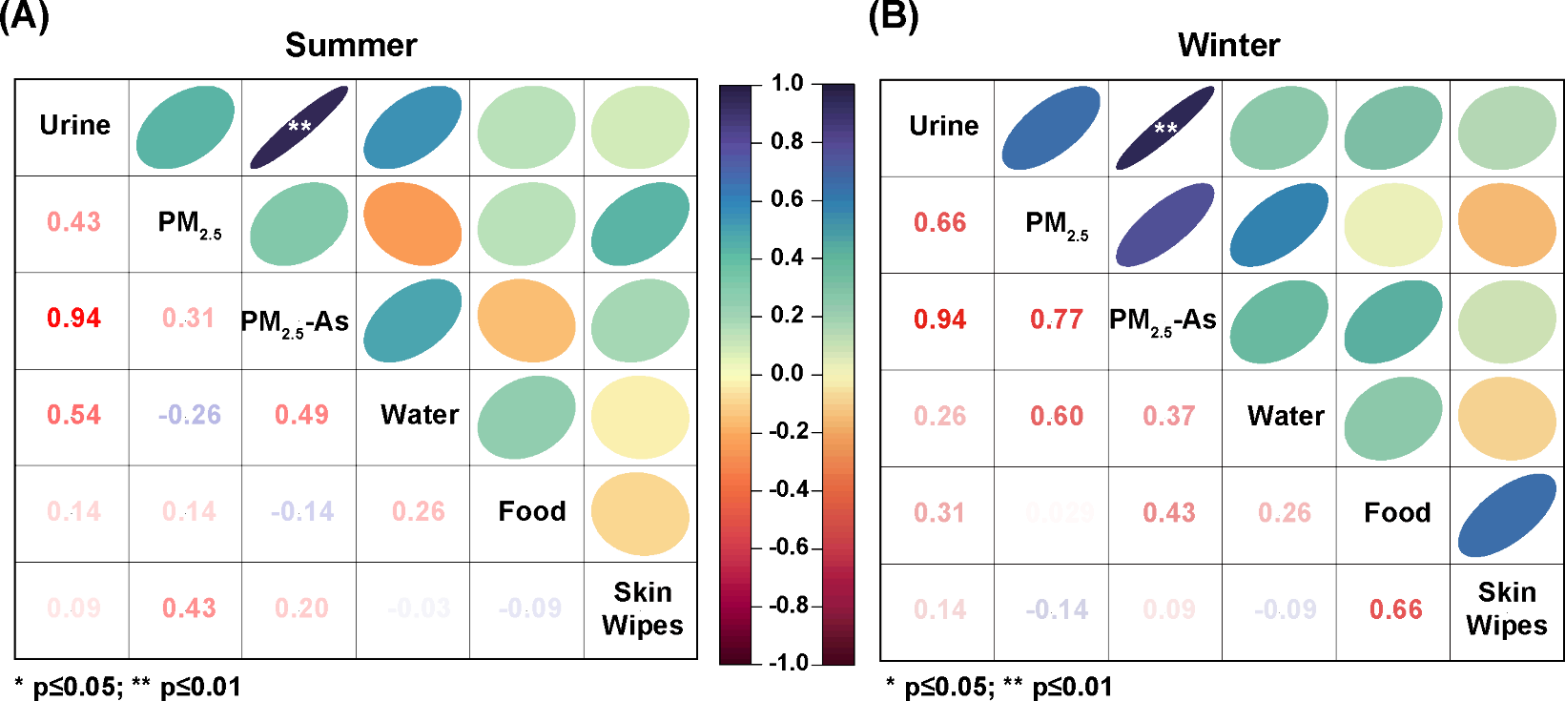
Correlation analysis was conducted between the urine samples of the workers and the concentrations of PM_2.5_, PM_2.5_-As, food, drinking water, and skin wipe papers. The analysis was performed separately for the summer (A) and winter (B) seasons.

In order to study the significant exposure routes of As on the workers at the smelting area, we carried out a cohort study (Table S6) among them to analyze the association between the daily tAs level in urine and the level of As at the main environmental media of the site, including PM_2.5_. Figure 2 shows that there is a strong correlation between the As concentration in urine (U–As) and PM_2.5_-As in both summer and winter (R^2^ = 0.94, both), confirming that PM_2.5_ is the predominant bridge (exposure media) for environmental As to enter the human body at smelting sites. Many previous studies have consistently demonstrated that ingestion is the primary route through which heavy metals enter the body at smelting sites.^30,31^ In contrast, there is no correlation between As concentration in diet and drinking water and As absorbed by workers. A possible explanation for this might be the enhanced food safety measures implemented in China’s nonferrous metal smelting industry.^32^ China has implemented a soil classification management system, which not only protects arable lands, but also prohibits the use of contaminated soil around nonferrous metal smelting site as cultivated land.^33^ China ’s e-commerce logistics supply chain system has developed rapidly in the past decade, which allows nonferrous metal smelting enterprises to purchase safe and high-quality food materials quickly and conveniently.^34^ In our previous site survey, we also found that most of the food materials of factory workers were obtained through online shopping. PM_2.5_ is defined as particles that are 2.5 μm or less in diameter, which is more likely to travel into and deposit on the surface of the deeper parts of the lung. With respect to exposure to PM_2.5_ types, individuals’ respiratory system is the major pathway enabling PM_2.5_ penetration in lung tissues. When the PM_2.5_ carry heavy metals, these metals can become attached or adsorbed to the surfaces of the particles. As a result, the heavy metals can be transported along with the PM_2.5_ particles into the respiratory system. Our results indicated that inhalation of PM_2.5_-As was the main route of As exposure on the workers at the smelting area.To the best of our knowledge, this is the first study to report such an association in a smelting area.

### Bioaccumulation, Speciation Translation and Bioavailability of PM_2.5_-As

**Figure 3:**
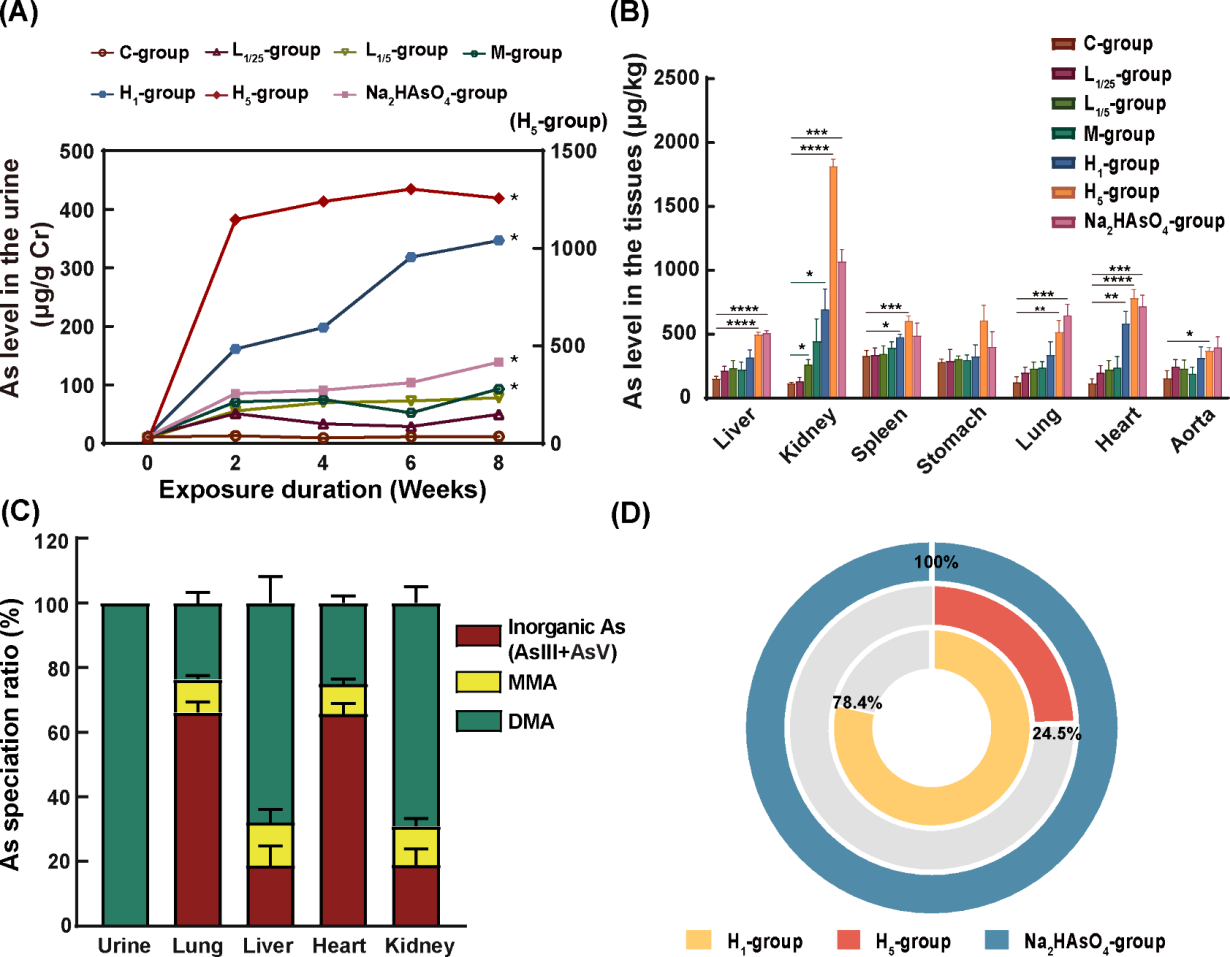
Bioaccumulation and chemical speciation analysis of PM_2.5_-As. (A) Trend diagram of arsenic content in urine. (B) Arsenic concentration in the tissues of mice in different exposure groups. (C) Chemical speciation ratio of As in the urine and tissues. (D) Average RBA of As in H_1_-, H_5_- and Na_2_HAsO_4_-groups. Values are mean ± SD of six mice. *p vs C group < 0.05, **p vs C grou*p* < 0.01, ***p vs C group < 0.001, ****p vs C group < 0.0001.

To estimate the internal exposure factors of PM_2.5_-As, such as bioaccumulation, distribution, and speciation transformation, an animal exposure study was conducted based on the environmental concentration, composition, and speciation of PM_2.5_-As. The exposure method and the groups utilized in the study are depicted in Figure S3. Figure 3A shows the change trend of As concentration in urine of mice during exposure, which is used to reflect the As absorption level of mice. Following 42 days exposure, the H_5_ group reached a stable state in terms of total U–As, suggesting that the amount of As taken by the mice and eliminated through metabolic processes had reached an equilibrium. After the exposure, with the exception of the L groups (L_1/25_ and L_1/5_ groups), the U–As concentration in the exposure group was significantly higher than that in the C group. The level of U–As has been widely used as an indicator of human exposure because urine is the main excretion route for most arsenic species.^35,36^ Our results not only showed a good linear relationship between the dosage from environmental inhalation of As and tAs in urine, but also confirmed the feasibility of our cohort study using U–As as a marker to track human As exposure levels. Additionally, inhalation exposure by mice to As for 8 weeks clearly increased the tAs level in the hair compared to the C group, which is similar to the results observed in the urine (Figure S4). This result indicated that As concentrations in hair can reflect both As exposure and intake concentrations, which may provide a more efficient tool for detecting and monitoring As exposure than the traditional method (blood and urine). The main component of hair is α-Keratin containing numerous sulphydryl groups, and As can form covalent complexes with sulphydryl groups and accumulate in hair for a long time.^37^ Since As concentrations along hair length reflect the time and intensity of exposure, hair samples can be used as markers to assess individual arsenic exposure over time.^38^ Previous studies also reported the close relationship between As concentrations in hair and exposure through various sources such as drinking water, contaminated soil, and dietary intake.^39−41^

The tissue/organ bioaccumulation and distribution pattern in the mice were examined following As inhalation exposure (Figure 3B). The order of As accumulation levels in the tissues of high-dosage mice was observed to be kidney > heart > spleen > stomach > liver > lung > aorta. The accumulation levels of As in the kidneys and spleens were high in the M group, whereas there was no significant difference in the L and C groups (Table S7 and Figure 3B). As an essential organ for excretion, the kidney is where As is methylated following entry into the body to create DMA, so the kidney has the highest concentrations of As.^42^ Additionally, the liver also represents a significant target of As bioaccumulation and methylation regulated by glutathione.^43^

Unsurprisingly, As buildup in the heart was also extremely high, so would undoubtedly raise the incidence of CVD. The speciation of As in different tissues of mice varied greatly after passing through the respiratory tract (Figure 3C, Table S8). In urine, only DMA was detected in the H_5_ group, which is considered as the final metabolite in mammals.^44^ At the same time, DMA and MMA are the dominant chemical forms in tissues and organs with strong catabolic ability such as the kidney (DMA:69.1%, MMA:12.0%) and liver (DMA:67.9%, MMA:13.4%). Notably, inorganic As (AsIII and AsV) with strong toxicity was also detected in the heart and lung, in proportions of 65.7% and 66.1% respectively. The above results indicated that, in the toxicological study of As exposure, more attention should be paid to tissues and organs with relatively weak metabolic transformation ability.

The As RBAs of kidney, hair and stomach for the H_5_ group were 34.0%, 30.9% and 30.4%, followed by spleen and heart, liver, aorta and lung at 24.8%, 21.8%, 19.5%, 18.6% and 16.0% respectively (Figure S5). The average tissue and hair bioavailability was 24.5% in the H_5_ group (Figure 3D). Although the bioaccumulation level of As in the H_5_ group was the highest in all exposure groups, the As RBA for the H_5_ group was the lowest of all exposure groups (H_5_ = 24.5%, H_1_ = 78.4%, M = 318%, L_1/5_ and L_1/25_ are much larger than M). In this study, RBA was selected to measure the inhalation bioavailability of PM_2.5_-As, which compared As accumulation in mice following exposure to PM_2.5_-As to that of a soluble reference NaH_2_AsO_4_. Therefore, the relatively low RBA in the H_5_ group may be related to its high exposure dose. Previous studies reported that the oral bioaccessibility values of As, obtained using *in vitro* extraction methods from mine area soil, in most cases were less than 30%, which is commonly lower than the As RBA values measured in animal studies.^19,45^ Theoretically, soluble arsenic compounds were essentially 100% bioavailable, which is consistent with our data for the L groups. However, the difference in bioavailability between different exposure concentration groups found in our study suggests that, during long-term exposure, the body’s ability to absorb, accumulate and secrete heavy metals is not immutable. Therefore, we suggest that the exposure mode and parameters should be selected according to the real environmental factors of the studied area in investigating the bioavailability of environmental pollutants.

### Chronic PM_2.5_-As Exposure Induced Cardiac Dysfunction, Oxidative Stress, and Inflammation

**Figure 4:**
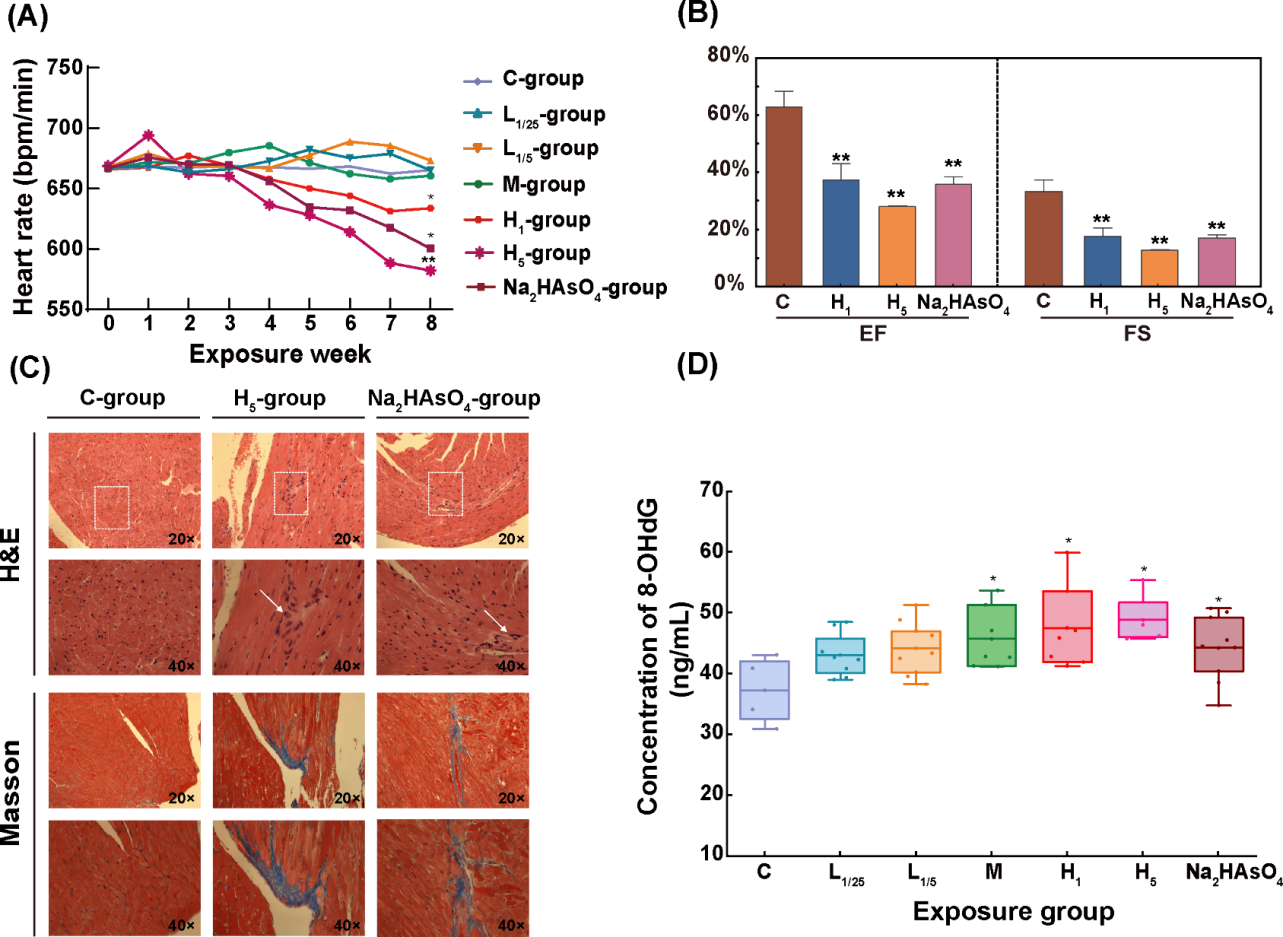
Cardiac injury and dysfunction induced by PM_2.5_-As exposure. (A) Average heart rate of each group of mice during exposure. (B) Comparison of EF and FS between M, H, and Na_2_HAsO_4_ groups and the C group. (C) Histopathological images of the hearts of mice from the C, H_5_, and Na_2_HAsO_4_ groups. (D) Concentration of 8-OHdG in the urine of mice in different exposure groups. Values are mean ± SD of six mice. *p vs C group < 0.05, **p vs C grou*p* < 0.01.

Most studies have shown that As exposure can induce or aggravate a number of CVDs.^6,46^ Therefore, based on the analysis of chemical speciation and bioavailability, we used the mice to investigate the damaging effects of PM_2.5_-As on the cardiovascular system. Mice’s HRs were recorded on a weekly basis (Figure 4A). H groups’ (H_1_ and H_5_ groups) and the Na_2_HAsO_4_ group’s HRs first rose and eventually fell. In particular, after around 3 weeks of exposure, they both started to drop collectively. The H_5_ group and Na_2_HAsO_4_ group both saw early reductions, with the H_5_ group showing the clearest fall. The HR in the M group exhibited an upward trend in the second week, reaching its peak in the fourth week, followed by a subsequent decline. Surprisingly, by the end of the exposure trial, it had returned to its initial level. This phenomenon was also observed in our previous PM_2.5_ exposure study.^18^ When the damage caused by external chemical substances is temporary or reversible, the body can repair the damage and recover related function by itself.^47,48^ There was no appreciable difference in HRs between the M group and L groups. Our previous study found that heart automaticity is an important environmental toxicological indicator of heart. The trend of HR changes in mice during PM_2.5_-As exposure in the H groups was basically consistent with the previous cardiac dysfunction induced by pollutants.^9,18^ The BP of all mice in the awake state was measured before the end of the exposure experiment (Figure S6). Compared with the C group (112/88.6 mmHg), diastolic blood pressure (DBP), systolic blood pressure (SBP), and pulse pressure (PP) significantly increased in the H_1_ (134/107 mmHg), H_5_ (145/103 mmHg) and Na_2_HAsO_4_ groups (127/105 mmHg), indicating typical vascular relaxation.

When significant anomalies in HR and BP were found during the eighth week of exposure, echocardiography was carried out to assess cardiac dysfunction in mice who may have experienced a cardiovascular injury (Figure S7). The cardiopulmonary function of mice was assessed using the ejection fraction (EF) and fractional shortening (FS) methods (Figure 4B). The C group had greater EF and FS values than those of the M, H, and Na_2_HAsO_4_ groups (EF: p= 0.0144, 0.0022, 0.0003 and 0.0016, respectively; FS: p= 0.0094, 0.0018, 0.0004 and 0.0015, respectively), with the levels of the C group very near to normal levels from our previous work.^49^ In conclusion, our results indicated that chronic exposure to excessive As could worsen cardiac dysfunction. Compared with the C group, the H&E staining results of mice in the H_5_ and Na_2_HAsO_4_ groups showed more inflammatory cell infiltration (Figure 4C). Additionally, Masson trichrome staining showed that the degree of cardiac fibrosis in the H_5_ and Na_2_HAsO_4_ groups was greater than that of the C group. Significantly elevated oxidative stress status marker (MDA, ROS) and decreased intracellular antioxidant marker (SOD) were also detected in the mouse hearts of H_5_ group (Figure S8). The shape of cardiomyocytes will degenerate, inflammatory cells will infiltrate more heavily, and the degree of fibrosis will grow as a result of chronic high concentrations of As, all of which enhance the risk of CVDs brought on by PM_2.5_-As.^50^

The level of 8-OHdG in urine reflects the degree of DNA oxidative damage in the body. Compared with office workers, the concentration of 8-OHdG in smelter workers was always higher (*p* < 0.0001) (Figure S9). Similarly, following an 8-week exposure to arsenic (As), the concentration of 8-OHdG in the urine of exposed mice was significantly higher compared to the C group (Figure 4D). High levels of 8-OHdG in urine are associated with atherosclerosis and heart failure, and our results also support this.^51^

Potential mechanisms of As-induced CVDs may involve oxidative stress, the impairment of nitric oxide and calcium homeostasis in the cardiac cells, metabolic disorder and altered mitochondrial and enzyme activity.^52,53^ Oxidative stress is a key driver of As-induced toxicity, leading to apoptosis and cardiac injury.^6,54^ As exposure induces heart damage through a signal pathway involving oxidative stress and inflammation. Arsenic-induced oxidative stress activates nuclear factor-kappa B (NF-κB), resulting in heart tissue inflammation.^55^ This inflammation, combined with ongoing oxidative stress, promotes cardiac tissue fibrosis and remodeling, contributing to heart damage. The precise mechanisms underlying cardiovascular toxicity caused by As exposure are not yet well-defined. Future research is needed to gain a comprehensive understanding of As-induced heart damage and dysfunction.

### PM_2.5_-As Health Risk Assessment

In order to assess the health risk of PM_2.5_-As in smelting sites more accurately, we first built up a health risk assessment model referring to the USEPA model, by integrating internal and external exposure parameters such as pollution characteristics, bioavailability and cardiovascular health effects. Considering the persistence and continuity of As exposure in the smelting site population, the stable RBA of the H_5_ group (24.5%) was selected to estimate the health effects of PM_2.5_-As exposure. USEPA has calculated a RfD of 3.01 × 10^–4^ mg/(kg·day) of oral As exposure,^56^ however, there is currently no available RfD or reference concentration (RfC) for chronic inhalation arsenic exposure. Therefore, we determined the BMDL of PM_2.5_-As in the smelting site based on the cardiovascular damage effect, and calculated the corresponding RfD (Text S7, Table S9 and Figure S10). The health risk RfD based on cardiovascular injury is 5.13 × 10^–5^ mg/(kg·day) and it is significantly lower than the USEPA risk value for skin and vascular effects associated with Blackfoot disease. Our RfD value further confirms the previous research results that the cardiovascular system is extremely sensitive to As exposure.^3,6^

The modified noncarcinogenic health risk assessment model through chronic inhalation PM_2.5_-As exposure with the cardiovascular system as the end point of toxic effect is given in eq 3:
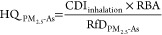
3where HQ_PM_2.5_ - As_ is the hazard quotient of PM_2.5_-As, RBA is the relative bioavailability of PM_2.5_-As (assumed to be the average RBA for the H_5_ group i.e. 24.5%), and RfD_PM_2.5_ - As_ is the reference dose of PM_2.5_-As, mg/(kg·day).

**Figure 5:**
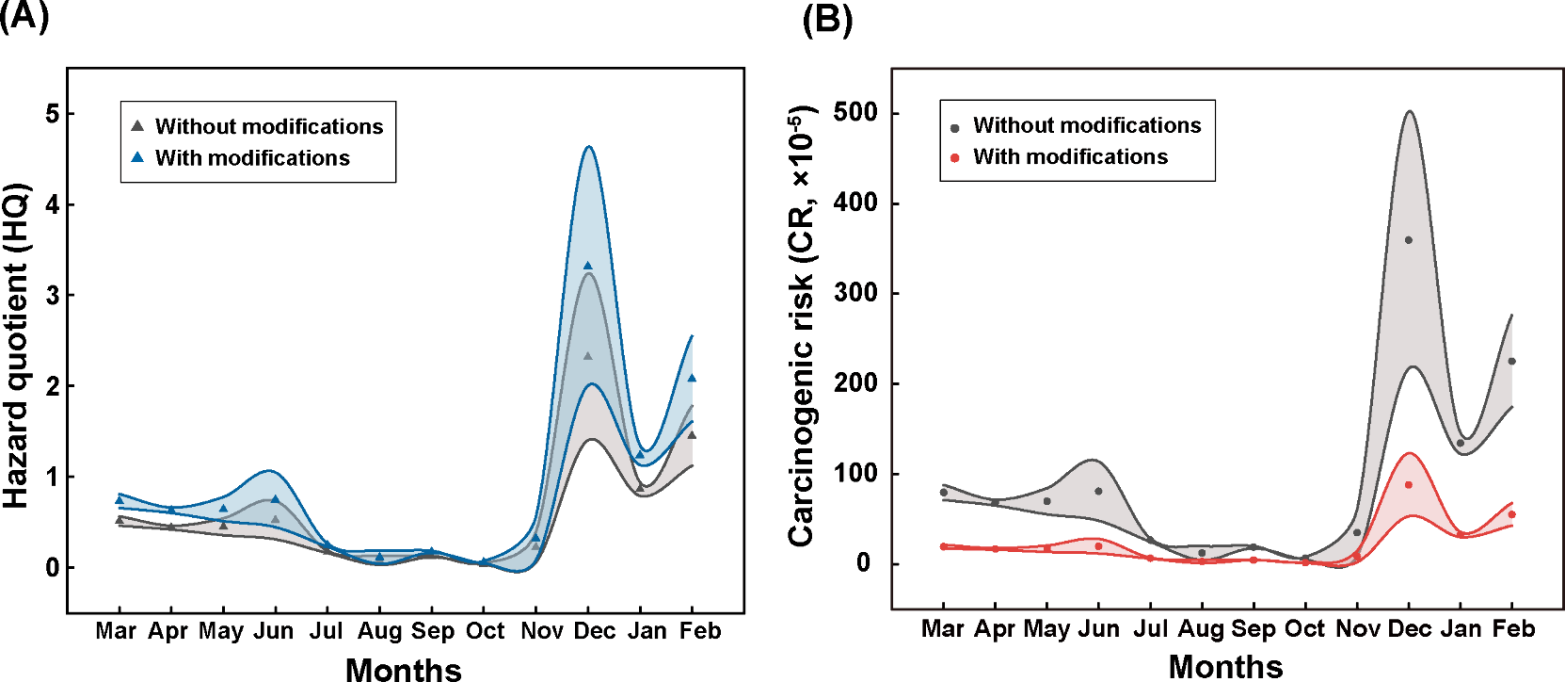
Comparison of HQ (A) and CR (B) calculated with and without modifications using the modified model.

As shown in Figure 5A, the cardiovascular-specific health risk is rather higher than the noncarcinogenic risk obtained from the USEPA health risk assessment. After implementing the modifications, December, January, and February all showed obviously noncarcinogenic risks to workers, associated with the PM_2.5_-As concentration (Figure 1A), and the lowest-observed-effect-concentration (LOEC) corresponding to the damage threshold calculation (0.64 μg/m^3^). This means that the previous model underestimates the harm of the smelting site to the cardiovascular system, the core system of the human body. Although we cannot determine that the cardiovascular system is the most sensitive end point of toxic effects of arsenic exposure, the health risk model established by combining cardiovascular toxicity with RBA will enable accurate noncarcinogenic risk assessment of workers at smelter sites exposed to PM_2.5_-As through inhalation.

Additionally, using the modified carcinogenic risk model (eq 4), the carcinogenic risk of PM_2.5_-As considering the RBA obtained from our studies was clearly lower than that without modification (Figure 5B). Without any modifications, the health risk of As exposure from PM_2.5_ was assessed only by external total PM_2.5_-As exposure dose, which may incorrectly estimate the health risk of As.
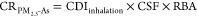
4where CR_PM_2.5_ - As_ is the carcinogenic risk of PM_2.5_-As, and CFS is the cancer slope factor, [(mg/(kg·day)]^−1^.

However, it is important to note that this approach does not account for additional exposure pathways, such as ingestion and dermal contact. Therefore, it is crucial to identify the primary exposure pathways, considering the diverse range of human exposure routes and individual variabilities. Moreover, more studies are needed to evaluate the health risks associated with various contaminants present in PM_2.5_ at integrated smelting facilities.

## Supporting Information Available

Chemical reagents and sampling information; ICP-MS and XPS detection conditions; quality assurance and quality control (QA/QC); exposure solution calculation and preparation; As concentrations in various tissues; characteristics of panel study participants; calculation process of RfD; fitted dose–effect relationship models based on the BMDS online (PDF)
