# Supplementary material for: Assessing the Impact of PM2.5-Bound Arsenic on Cardiovascular Risk among Workers in a Non-ferrous Metal Smelting Area: Insights from Chemical Speciation and Bioavailability
Source: Environ Sci Technol. 2024 May 2;58(19):8228–38. doi: 10.1021/acs.est.3c10761 (PMC11097390; doi:10.1021/acs.est.3c10761)
Supplement: Supplementary file 1 — es3c10761_si_001.pdf [file es3c10761_si_001.pdf]

Supporting Information for

Assessing the Impact of $PM_{2.5}$-Bound Arsenic on Cardiovascular Risk Among Workers in a Non-Ferrous Metal Smelting Area: Insights from Chemical Speciation and Bioavailability

Zenghua Qi[a], Qiting Zhao[a], Zixun Yu[a], Zhu Yang[b], Jie Feng[a], Pengfei Song[a], Xiaochong He[a], Xingwen Lu[a], Xin Chen[c], Shoupeng Li[d], Yong Yuan[a], Zongwei Cai[a,b*]

Contents:

Supplementary text

Text S1. The specific methods of preprocessing and pre-detection for filter membranes

Text S2. Quality assurance and quality control (QA/QC)

Text S3. XPS detection parameter (XPS) analysis process

Text S4. Chemical reagents

Text S5. Solution preparation in animal experiment

Text S6. The procedure of hematoxylin and eosin (H&E) staining and Masson staining

Text S7. The calculation process of RfD

Table S1 – Table S9

Table S1. Microwave digestion temperature program

Table S2. ICP-MS detection conditions

Table S3. XPS detection parameter

Table S4. Simulated $PM_{2.5}$ drip suspension configuration

Table S5. Comparison of the $PM_{2.5}$-As value between the study site and national city in China

Table S6. Characteristics of Panel Study Participants

Table S7. Concentrations (mg/g, Dry Weight, dw) of Arsenic in Various Tissues of C57BL/6 Mice

in Five Exposure Groups (Mean ± SD)

Table S8. The concentrations of different arsenic species (ng / g, wet weight, dw) in different tissues of C57BL/6 mice in the H_5 highest concentration arsenic exposure group.

Table S9. The fitted dose-effect relationship model and the BMD-CI based on the EPA Benchmark Dose Tools (BMDS) Online

Figure S1 – Figure S10

Figure S1. The distribution of sampling points at the non-ferrous metal smelting site

Figure S2. As concentration in the soil, dust, and tailings in the factory and surrounding areas

Figure S3. Animal exposure and physiological index collection process design.

Figure S4. Arsenic concentration in the hair of mice in different exposure groups.

Figure S5. Bioavailability of $PM_{2.5}$-As in different tissues of mice.

Figure S6. Blood pressure of mice in each group at the eighth week of exposure. The white part is diastolic blood pressure (DBP), the colored part is systolic blood pressure (SBP), and the oblique part is pulse pressure (PP).

Figure S7. Echocardiography of mice in C, M, H, and Na_2HAsO_4 groups.

Figure S8. Concentration of 8-OHdG in the urine of workers in the smelting area.

Figure S9. Effects of $PM_{2.5}$-As exposure on the ROS, MDA and SOD production of mice in H_5 group.

Figure S10. The fitted dose-effect relationship models based on the BMDS online.

Supplementary Text

Text S1. The Specific Methods of Preprocessing and Pre-detection for Filter Membranes.

A technique that makes use of filter collection—the gravimetric analysis—was used to confirm the mass concentration of $PM_{2.5}$. Prior to and after the sampling procedure, the weight of each filter membrane was measured with a precision of 0.01 mg using a microelectronic scale. In the end, the result was obtained by averaging three parallel datasets and then using the following formula to calculate the average.

$$C = \frac{(W_2 - W_1) \times 10^{-6}}{V_0} \quad (1)$$

where C stands for the mass concentration of $PM_{2.5}$ in the atmosphere (μg/m^3), W_1 stands for membrane quality before sampling (g), W_2 stands for membrane quality after sampling (g), V_0 is the sampling volume converted by standard conditions (m^3).

We further treated the filter membrane to ascertain the primary anion content in $PM_{2.5}$. Similar to the previous study, ion chromatography was used to identify the eight water-soluble inorganic ions (K^+, Ca^{2+}, Na^+, Mg^{2+}, Cl^-, SO_4^{2-}, NO_3^-, and NH_4^+) in the supernatant following ultrasonic extraction in a water bath at 70°C for three hours. The total carbon analyzer provided a quantitative estimate of the amount of elemental and organic carbon[1].

Text S2. Quality Assurance and Quality Control (QA/QC)

The many repeats idea was used in ICP-MS detection. The quantitative analysis approach with internal standard adjustment was employed to make the determination. It was promised that each sample, which included human pee, skin wipes, food, drinking water, mouse tissue, human urine, and other samples, would be repeated three times and identified three times. The same filter blank, reagent blank, and high recovery rate conditions were used for all sample digestion operations. To guarantee that three replications of the same sample were obtained after digestion, all samples employ the same rinse process, transfer method, digestion power, and digestion time (Table S1).

A single-tube peristaltic pump was used to pump the digestion solution for each sample into the atomization system, where it was then exposed to the plasma torch flame. The peristaltic pump

was cleaned for two minutes prior to the subsequent sample injection using pure water and 2% nitric acid before each detection. The ICP-MS instrument's operating guidelines were followed, and it was set up to function at its best. The necessary settings were made, and the peak-hopping mode was employed to collect the data. Table S2 displays the precise parameters. The standard solution, blank solution, and sample solution may be found in that order after the instrument is steady.

The accuracy was 3% and the recovery was 95.0%. To guarantee the accuracy of the experimental data, the relative percentage difference of the parallel samples was kept to a maximum of 20.0%.

Text S3. XPS Detection Parameter Analysis Process

We performed the XPS studies in order to determine the content and the chemical state of arsenic in $PM_{2.5}$ samples. XPS analysis was carried out on the Escalab 250Xi spectrometer (ThermoFisher, US) with an Al Kα X-ray source (1486.6 eV photons) and a pass energy setting of 20 eV under an ultrahigh vacuum. The spectra were corrected using C (1s) from contamination at 284.6 eV, and the observed spectra were fitted by the program of Advantage. The XPS detection parameters are shown in Table S3.

Text S4. Chemical Reagents

Standard solutions of arsenite (As III) and arsenate (As V) were purchased from O2Si (Charleston, SC, U.S.A). A standard solution of monomethyl arsenic Acid (MMA) was purchased from the China Institute of Metrology. Sodium arsenate heptahydrate ($Na_2HAsO_4 \cdot 7H_2O$) was purchased from Sigma-Aldrich (St. Louis, MO, U.S.A). Nitric acid (HNO_3, purity 65%) and hydrochloric acid (HCl, purity 30%) were purchased from Guangzhou Chemical Reagent Factory (Guangzhou, China). Ascorbic acid was purchased from Aladdin (Shanghai, China) and thiourea was purchased from Macklin (Shanghai, China). Normal saline (NaCl, purity 0.9%) was purchased from Kelun Pharmaceutical Co., Ltd. (Sichuan, China). Reagents and standards were manufactured and diluted using ultrapure water, which was obtained from the water purification system of Haokang Co. Ltd. (Chengdu, China).

Text S5. Solution Preparation in Animal Experiment

The intermediate solutions of 20 ppm and 100 ppm were made, respectively, and then diluted in accordance with the concentration of each group. The standard solution was mixed in accordance with the proportion of different chemical speciation in $PM_{2.5}$-As. Table S4 displays the particular setup concentration.

Text S6. The Procedure of Hematoxylin and Eosin (H&E) Staining and Masson Staining

While some newly taken samples from each group were maintained at -80°C by immediately transferring them into liquid nitrogen for tAs detection, other samples were selected at random and placed in paraformaldehyde fixative for later tissue slicing, Masson staining, and hematoxylin and eosin (H&E) staining. Cell images were taken with a Leica Micro Systems Inc., USA, DM6B upright microscope that has 20 and 40 objectives. Image-Pro Plus 7.0 (Media Cybernetics, Rockville, MD, USA) was used to analyze the fibrosis regions in the control and treated groups.

Text S7. The Calculation Process of RfD

We evaluated the cardiovascular damage by measuring heart rate, blood pressure, urinary 8-OHdG level, and the As content of tissue and urine in mice, where we took the last week of data to assess the final exposure effect. Table S8 and Figure S4 displayed the fitted dose-effect relationship models and the BMD confidence intervals (BMDL and BMDU). The calculated BMDL and BMDU values were 1.54 μg/kg and 3.66 μg/kg, respectively. The BMDU/BMDL ratio in our study was 2.38, which was less than 20 and suitable for effective BMD measurement[2]. As a result, we derived a point of departure (POD) value for $PM_{2.5}$-As by using the BMDL. The computed POD value, 0.64 $\mu g/m^3$, served as the threshold dosage for workers exposed to $PM_{2.5}$-As compounds in smelting areas.

The human body may suffer harm if the atmospheric concentration of $PM_{2.5}$-As exceeds this level. USEPA has suggested a reference dose (RfD) of 3.0×10^{-4} $mg\cdot kg^{-1}\cdot d^{-1}$ for chronic oral As exposure, however, there is currently no available RfD/RfC for chronic inhalation arsenic exposure. Then RfD is calculated by BMDL with formula (2)[3, 4].

$$RfD_{PM_{2.5}-As} = \frac{BMDL}{UF} \qquad (2)$$

where $RfD_{PM_{2.5}\text{-}As}$ is the reference dose of $PM_{2.5}$-As ($mg \cdot kg^{-1} \cdot day^{-1}$), BMDL is the benchmark dose limit ($mg \cdot kg^{-1} \cdot day^{-1}$), UF is the uncertainty factor (set at 30, of which the population variability factor was 10, and the pharmacodynamic component factor of inter-species variability was 3).

Table

Table S1. Microwave digestion temperature program.

Step	Heating-up time (min)	Target temperature (℃)	Holding time (min)
1	10	100	2
2	10	150	3
3	10	180	25

Table S2. ICP-MS detection conditions.

RF Power	Sampling Depth	RF Matching	Auxiliary flow	Carrier Gas	Omega Bias	Omega Lens	Cell Entrance	Deflect
1.55kW	5.0 mm	1.80V	0.80L/min	1.00L/min	-105V	10.1V	-45.0V	3.80V

Table S3. XPS detection parameter.

Source Gun Type	Spot Size	Lens Mode	Analyzer Mode	Energy Step Size	Number of Energy Steps	Number of Scans
Al K Alpha	650μm beam spot	Standard	CAE: Pass Energy 20.0 eV	0.050 eV	441	10

Table S4. Simulated $PM_{2.5}$ drip suspension configuration.

	Na_2HAsO_4-group	$L_{1/25}$-group	$L_{1/5}$-group	M-group	H_1-group	H_5-group
The exposure dose of simulated $PM_{2.5}$-As per mouse [μg/(kg·b.w.)]	8.51	0.06	0.31	1.57	8.51	42.5
Concentration of drip suspension (mg/L)	12.8	0.09	0.47	2.36	12.8	63.8

Table S5. Comparison of the $PM_{2.5}$-As value between the study site and national city in China.

**Study area**	**Content of $PM_{2.5}$-As (μg/m³)**
Northern China	0.008
Southern China	0.05
Eastern China	0.02

Study area	Content of $PM_{2.5}$-As (μg/m³)
Central China	0.04
Southwestern China	0.007
Northeastern China	0.01
Northwestern China	0.02
National Average	0.02
Average content measured in smelt site	0.74

Table S6. Characteristics of panel study participants.

Characteristic	Frequency (%)	Mean (SD)	Median (Range)
Profession			
Worker	25 (100)		
Sex			
Male	13 (52)		
Female	12 (48)		
Age, years		43.5 (7.22)	44.0 (25.0-57.0)
Length of local residence, years			
1-5	2.0 (8.0)		
>10	23 (92)		
Smoking			
Yes	5.0 (20)		
No	20 (80)		
Drinking alcohol			
Yes	5.0 (20)		
No	20 (80)		
Heart rate, bpm		78.0 (10.0)	77.0 (66.0-106)
Blood pressure			
Systolic pressure, mmHg		129 (9.00)	128 (103-154)
Diastolic pressure, mmHg		84.0 (7.00)	84.0 (74.0-110)

Table S7. Concentrations (mg/g, Dry Weight, dw) of arsenic in various tissues of C57BL/6 mice in five exposure groups (Mean ± SD).

	Tissue					
	Sleep	Liver	Kidney	Heart	Lung	Stomach
M-group	0.59±0.03	15.9±0.27	3.48±0.18	0.97±0.73	1.24±0.01	3.61±2.40
H_1-group	0.64±0.03	14.9±0.13	3.62±0.33	1.05±0.06	1.27±0.02	1.90±0.28
H_5-group	0.72±0.13	14.9±0.97	3.51±0.12	1.38±0.04	1.11±0.05	1.97±0.08
Na_2HAsO_4 -group	0.63±0.07	17.2±0.20	3.90±0.03	1.31±0.12	1.20±0.05	2.88±0.70
C-group	0.65±0.03	13.3±0.37	3.37±0.19	1.25±0.15	0.99±0.14	2.44±0.40

Table S8. The concentrations of different arsenic species (ng/g, wet weight, dw) in different tissues of C57BL/6 mice in the H_5 group.

Tissue	DMA (ng/g)	As^{3+} (ng/g)	MMA (ng/g)	As^{5+} (ng/g)
**Liver**	16.7±3.66	NA	3.21±0.78	4.46±1.28
**Heart**	86.5±6.70	142±33.8	32.1±4.41	88.8±12.0
**Kidney**	19.1±3.73	NA	3.32±0.86	5.18±1.46
**Lung**	90.4±8.53	165±32.6	39.7±5.85	91.6±0.94

Table S9. Based on the USEPA Benchmark Dose Tools (BMDS) Online, the fitted dose-effect relationship model and the BMD-CI.

Effect	Recommended model	BMDL (μg/kg)	BMD (μg/kg)	BMDU (μg/kg)	BMDU/BMDL
UAs Content	Linear	1.18	1.42	1.75	1.48
Tissue As Content	Hill	1.24	1.82	3.21	2.60
Blood Pressure	Hill	0.38	0.73	0.75	1.96
Heart Rate	Hill	3.36	8.74	8.92	2.65
8-OHdG Content	Hill	0.01	0.06	2.64	264
**Average**	--	1.54	3.18	3.66	2.38

Figure

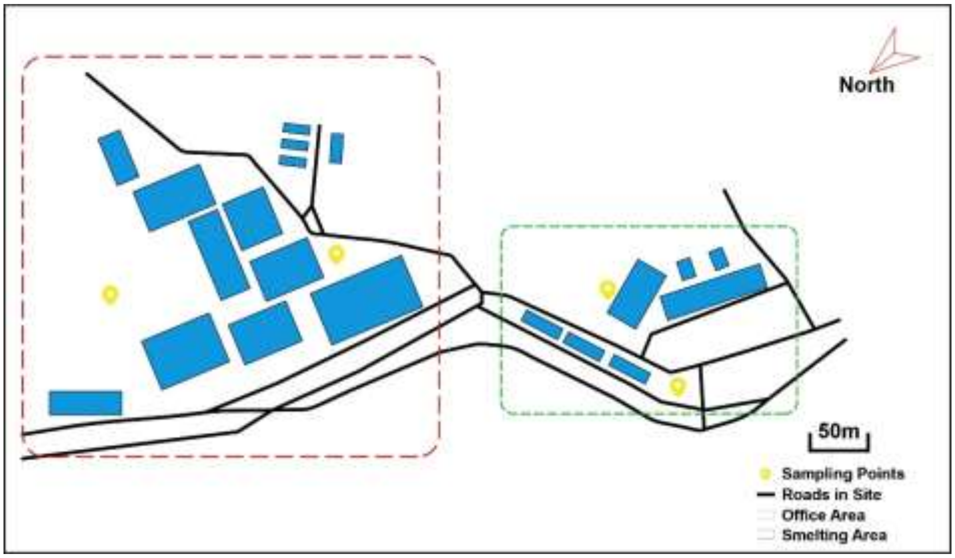

Figure S1. The distribution of sampling points at the non-ferrous metal smelting site.

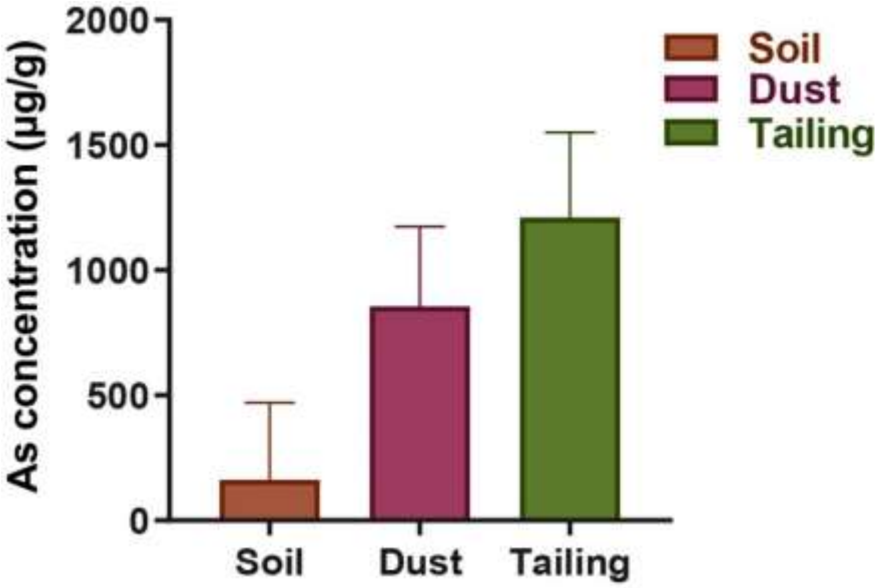

Figure S2. As concentration in the soil, dust, and tailings in the factory and surrounding areas.

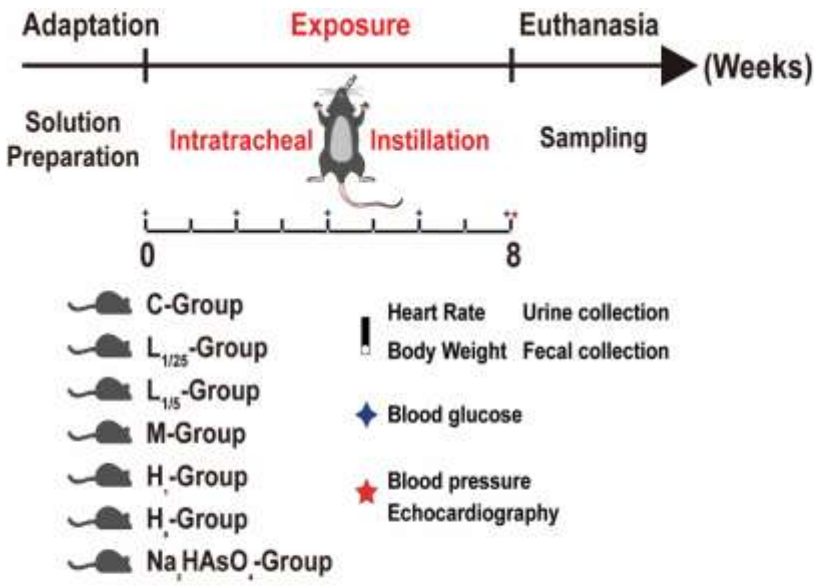

Figure S3. Animal exposure and physiological index collection process design.

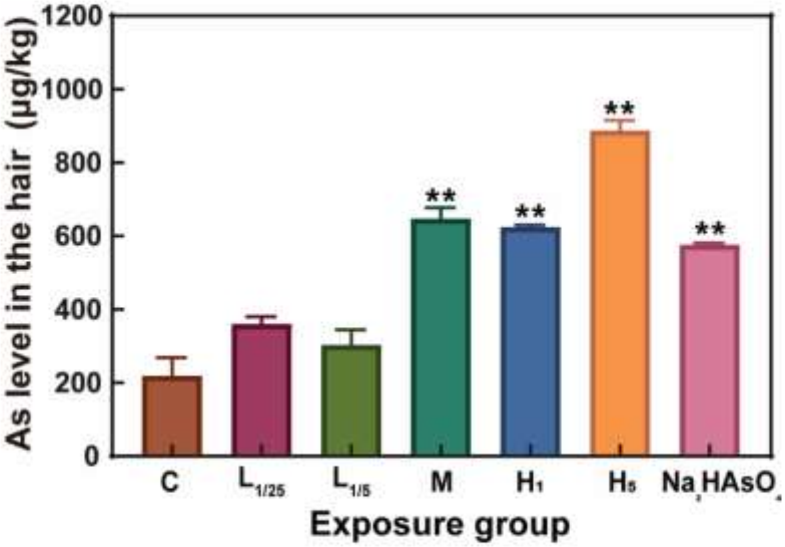

Figure S4. Arsenic concentration in the hair of mice in different exposure groups.

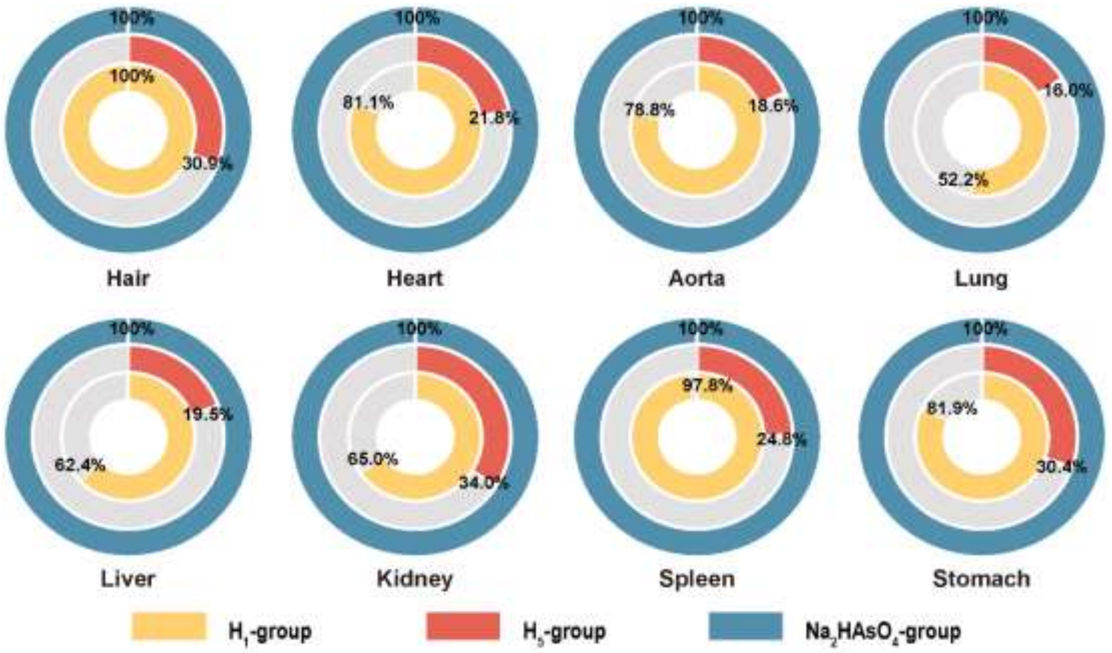

Figure S5. Bioavailability of $PM_{2.5}$-As in different tissues of mice.

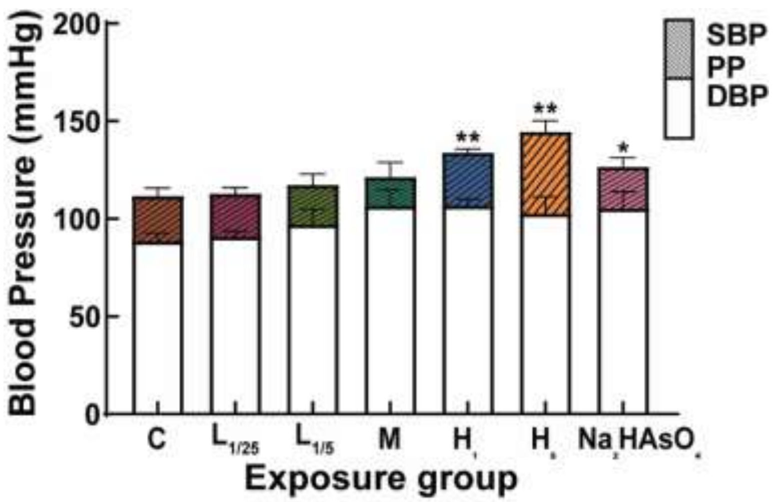

Figure S6. Blood pressure of mice in each group at the eighth week of exposure. The white part is diastolic blood pressure (DBP), the colored part is systolic blood pressure (SBP), and the oblique part is pulse pressure (PP).

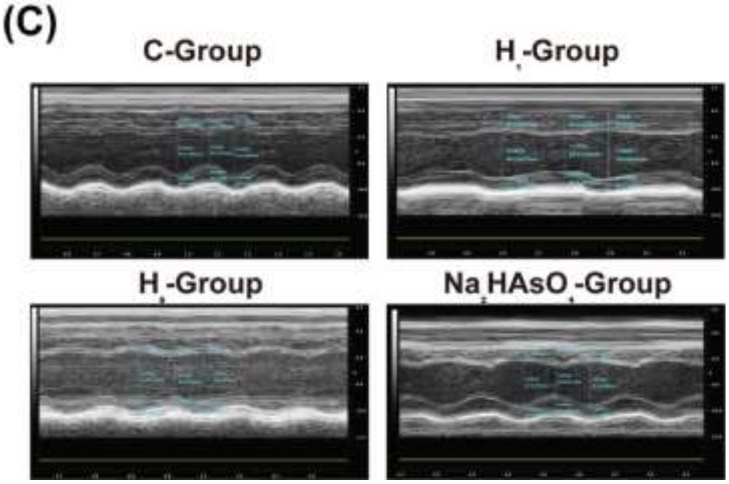

Figure S7. Echocardiography of mice in C, M, H, and Na2HAsO4 groups.

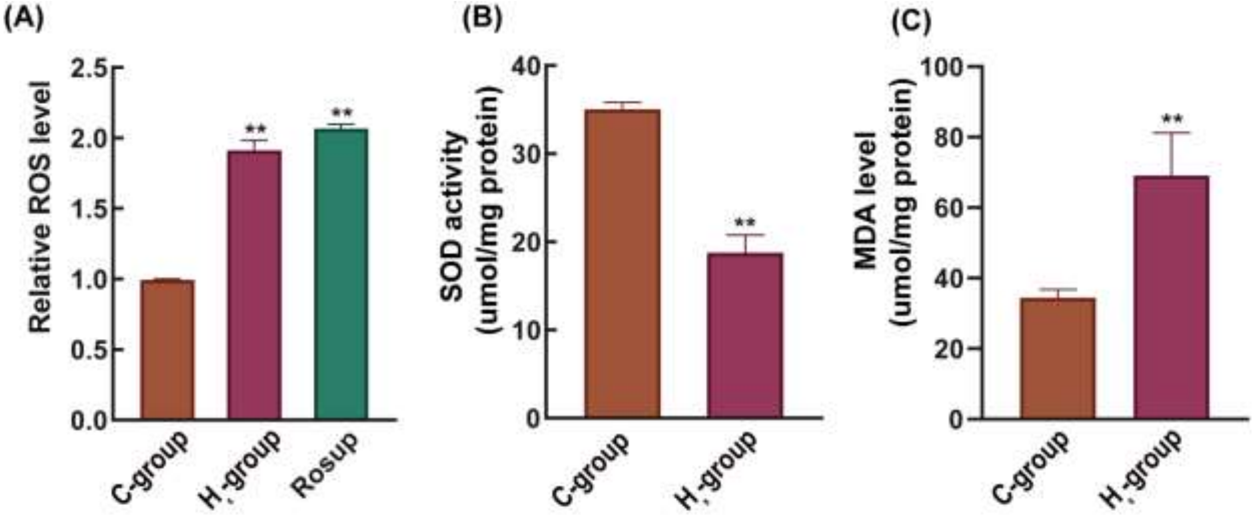

Figure S8. Effects of $PM_{2.5}$-As exposure on the ROS, MDA and SOD production of mice in H_5 group. The relative ROS levels (A) SOD activity (B) and MDA activity (C) levels in heart tissues compared to control. Data are calculated as fold changes compared with control group and presented as mean ± SD (n=6) of three independent experiments. ***p* vs C group < 0.01.

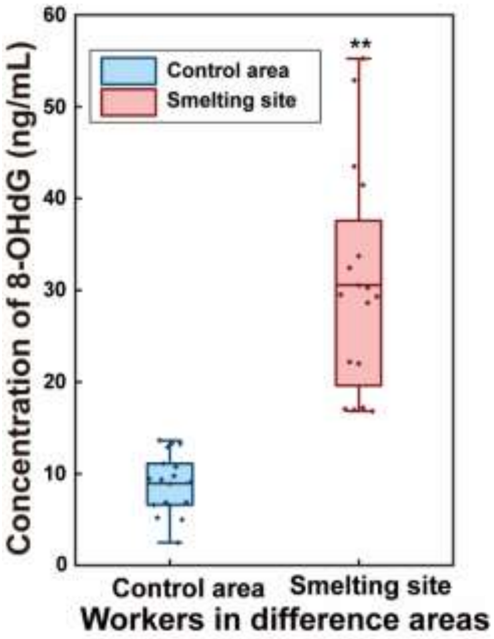

Figure S9. Concentration of 8-OHdG in the urine of workers in the smelting area.

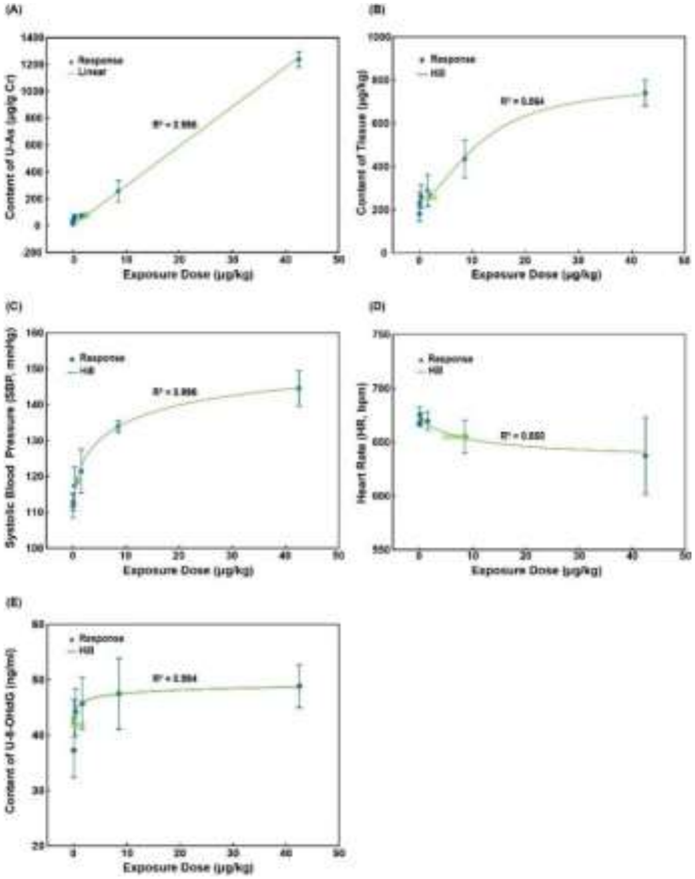

Figure S10. The fitted dose-effect relationship models based on the BMDS online.

1. Wen, L.; Yang, C.; Liao, X.; Zhang, Y.; Chai, X.; Gao, W.; Guo, S.; Bi, Y.; Tsang, S. Y.; Chen, Z. F.; Qi, Z.; Cai, Z., Investigation of PM(2.5) pollution during COVID-19 pandemic in Guangzhou, China. *J Environ Sci (China)* **2022,** *115*, 443-452.
2. Haber, L. T.; Dourson, M. L.; Allen, B. C.; Hertzberg, R. C.; Parker, A.; Vincent, M. J.; Maier, A.; Boobis, A. R., Benchmark dose (BMD) modeling: current practice, issues, and challenges. *Crit Rev Toxicol* **2018,** *48*, (5), 387-415.
3. Chen, Q.; Chou, W. C.; Lin, Z., Integration of Toxicogenomics and Physiologically Based Pharmacokinetic Modeling in Human Health Risk Assessment of Perfluorooctane Sulfonate. *Environ Sci Technol* **2022,** *56*, (6), 3623-3633.
4. Hindelang, P.; Scharinger, A.; Richling, E.; Walch, S. G.; Lachenmeier, D. W., Using the BMD Approach to Derive Acceptable Daily Intakes of Cannabidiol (CBD) and Tetrahydrocannabinol (THC) Relevant to Electronic Cigarette Liquids. *Front Biosci (Landmark Ed)* **2022,** *27*, (8), 228.
